# Mandibular Asymmetry, Generalized Joint Hypermobility, and Temporomandibular Disorders in Pre-Orthodontic Growing Individuals: A Cross-Sectional Clinical–Radiographic Study

**DOI:** 10.3390/jcm14175990

**Published:** 2025-08-25

**Authors:** Adriana Assunta De Stefano, Leda Miriam Valentini, Ludovica Musone, Martina Horodynski, Monica Macrì, Gabriella Galluccio

**Affiliations:** 1 Department of Oral and Maxillofacial Sciences, Sapienza University of Rome, 00161 Rome, Italy; adriana.destefano@uniroma1.it (A.A.D.S.); ledavalentini@outlook.it (L.M.V.); ludovicamusone@gmail.com (L.M.); gabriella.galluccio@uniroma1.it (G.G.); 2 Department of Innovative Technologies in Medicine & Dentistry, Università degli Studi “G. d’Annunzio” Chieti—Pescara, 66100 Chieti, Italy; monica.macri@unich.it

**Keywords:** temporomandibular disorders, joint instability, facial asymmetry, child, adolescents, cross-sectional studies

## Abstract

**Objective:** This study aims to explore the relationship between mandibular asymmetry (MA), generalized joint hypermobility (GJH), and temporomandibular disorders (TMD) in pre-orthodontic growing individuals. **Methods:** This cross-sectional study included 74 pre-orthodontic individuals aged 8–16 years. Mandibular asymmetry was evaluated through posteroanterior cephalometric analysis, using menton deviation ≥ 4 mm as the threshold for asymmetry. GJH was assessed using the Beighton Score (BS ≥ 4 = GJH-positive), while TMD was diagnosed based on Axis I of the DC/TMD. Associations among the variables were tested using a chi-square test (*p* < 0.05; SPSS v.24). **Results:** The study included 74 patients (25.7% males; mean age 12.7 ± 2.16 years). The GJH-positive group (*n* = 41) showed a higher prevalence of TMD (85.4%) compared to the GJH-negative group (51.5%) (*p* = 0.002). MA was more frequent in the GJH-positive group (68.3% vs. 45.5%; *p* = 0.041). A significant association was also found between TMD and MA (71.2% vs. 27.3%; *p* < 0.001). In both groups, patients with TMD were more likely to present MA (GJH-negative *p* = 0.022; GJH-positive *p* = 0.046). **Conclusions:** MA emerged as a key factor associated with the presence of TMD, particularly when combined with GHJ. These findings indicate that MA alone is significantly related to the occurrence of TMD, regardless of joint hypermobility status. However, the risk appears to be amplified in individuals who also present with GJH. Effect size analysis indicated that most associations were small, with only the one between TMD and MA reaching a moderate level. This highlights the importance of evaluating statistical significance in the context of effect size to better assess clinical relevance.

## 1. Introduction

Temporomandibular disorders (TMDs) are a group of musculoskeletal and neuromuscular conditions affecting the temporomandibular joint (TMJ), masticatory muscles, and associated structures [1]. These disorders can manifest with a wide range of symptoms, including joint sounds, pain in the orofacial region, masticatory dysfunction, and limitations in jaw movement [2,3]. Although TMDs are frequently studied in adults, growing evidence suggests that children and adolescents are also susceptible, particularly during the period of rapid craniofacial development [4,5,6,7,8,9,10,11].

The diagnosis of temporomandibular disorders (TMDs) typically involves a comprehensive clinical assessment, supported by imaging modalities such as radiography or magnetic resonance imaging (MRI), and, in some cases, laboratory investigations [12,13,14,15]. The prevalence of TMDs in pediatric and adolescent populations remains insufficiently characterized. This is primarily due to the infrequent diagnosis of TMD within these age groups and the absence of a universally accepted diagnostic definition specific to this demographic. Several factors contribute to this knowledge gap. Firstly, TMD is often under-recognized in children and adolescents, partly because it is not routinely considered a pediatric condition, and partly due to the low rate of healthcare-seeking behavior for TMD-related symptoms among younger individuals. Secondly, the lack of standardized diagnostic criteria across studies results in methodological heterogeneity, thereby limiting the comparability of prevalence estimates [5].

The Research Diagnostic Criteria for Temporomandibular Disorders (RDC/TMD), introduced in 1992, marked a significant step toward standardized evaluation of TMD by adopting a dual-axis system grounded in the biopsychosocial model. This approach allowed for both clinical assessment of physical symptoms (Axis I) and evaluation of psychosocial factors and pain-related disability (Axis II) [1]. Building on this foundation, the Diagnostic Criteria for TMD (DC/TMD), introduced in 2014, expanded the diagnostic framework by incorporating updated instruments for the assessment of psychological and behavioral dimensions, offering both brief screening tools and comprehensive evaluation protocols. Developed by an international team of clinicians and researchers, the DC/TMD provides a systematic and multidisciplinary approach to diagnosing TMD. It emphasizes the integration of detailed medical history and clinical examination, imaging techniques such as radiographs or magnetic resonance imaging to identify structural or degenerative changes in the temporomandibular joint, psychological testing to explore potential emotional or behavioral contributors to the condition, and laboratory tests to rule out systemic diseases that may present with overlapping symptoms. While the DC/TMD offers a structured and validated framework, clinical judgment remains essential, and diagnostic conclusions should be drawn by combining findings from multiple sources [5,6]. The diagnostic guidelines proposed by the American Academy of Orofacial Pain (AAOP) for temporomandibular disorders encompass a range of clinical signs, including pain in the jaw and facial regions, restricted mandibular function, joint sounds, episodes of joint locking, bruxism, headaches, and temporomandibular joint instability. However, these criteria were primarily designed for adult populations and may not adequately reflect the clinical manifestations observed in pediatric and adolescent patients, whose symptomatology can differ significantly. Existing TMD diagnostic criteria are sometimes used in pediatric populations, but their suitability remains unclear. In 2024, adaptations of the DC/TMD for children and adolescents were introduced. These recommendations include AXIS I and AXIS II and were developed for clinical and research use [16,17]. Further research is needed to assess the accuracy and consistency of existing diagnostic tools for pediatric TMD. Creating age-appropriate criteria is crucial for precise diagnosis and effective treatment, which can enhance outcomes and quality of life for affected children and adolescents [5,6,9].

The aetiology of TMD is considered multifactorial and complex, involving anatomical, functional, psychological, and systemic components [1,18]. Among the structural factors, facial asymmetry has been implicated as a potential contributor to the onset of TMD [19,20,21,22]. Facial asymmetry can be described as a difference in shape, size, or position between the structures on the sides of the mid-sagittal plane of the craniofacial complex [23,24]. A slight degree of facial asymmetry can be found in everyone [24,25] and it is defined as physiological asymmetry [26] to distinguish it from pathological asymmetries in which one of the craniofacial structures markedly deviates towards one side, causing functional and aesthetics problems [27]. Facial asymmetry, in particular mandibular asymmetry (MA), has been widely investigated in literature [24,28,29,30,31], since its shape and position often cause a non-symmetrical development of the facial complex [30,31]. MA can alter the biomechanical equilibrium of the stomatognathic system, possibly leading to compensatory muscle activity, joint overloading, and progressive dysfunction of the TMJ [24,28,29,30,31]. Despite this theoretical framework, the relationship between MA and TMD remains incompletely understood, especially in younger populations where asymmetry may be part of ongoing development.

Another factor of growing interest in relation to TMD is generalized joint hypermobility (GJH), a condition characterized by an excessive range of motion in multiple joints due to increased ligamentous laxity [32,33]. GJH is relatively common in children and adolescents and has been associated with various musculoskeletal complaints, including joint pain, instability, and fatigue [34,35]. Joint stability is granted by capsule tension and ligament holding; for these reasons, ligamentous laxity is often a cause of GJH. Joint laxity and the consequent joint hypermobility can also affect the temporomandibular joint, causing inflammation and intra-articular disorders [36].

The intersection of MA and GJH in relation to TMD remains an underexplored area. It is plausible that mandibular asymmetry, when occurring in a hypermobile systemic environment, may amplify the biomechanical stress on the TMJ and increase the risk of dysfunction [19,35,37]. Therefore, the aim of this study is to evaluate the relationship between MA, GJH, and the presence of TMD in a sample of growing individuals. Specifically, we seek to determine whether MA alone, or in combination with GJH, is associated with a higher prevalence of TMD. We hypothesize that growing individuals presenting both MA and GJH exhibit a higher prevalence of TMD compared to those with either condition alone or neither condition, as the combination of MA and GJH may represent a synergistic risk factor for the development of TMD. This investigation may provide new insights into structural factors contributing to TMD and help guide clinical assessments in at-risk populations.

## 2. Materials and Methods

### 2.1. Study Population

This observational cross-sectional study was conducted over a 12-month period from February 2024 to February 2025 through the observation of patients presenting for orthodontic evaluation at the Orthodontic Unit (UOC) of Policlinico Umberto I in Rome. Patients of both sexes, aged between 8 and 16 years, were included if they provided complete personal and medical history data, along with informed consent for the use of their clinical records for research purposes. Exclusion criteria included the presence of mandibular displacement due to occlusal precontacts, syndromic or congenital craniofacial asymmetries, a history of craniofacial trauma, or previous orthodontic treatment.

The study complied with the Helsinki Declaration’s guidelines for human research protocol and was approved by the Institutional Ethics Committee of Policlinico Umberto I (N.47/19/0001155). Written informed permission forms were collected from parents who agreed to participate after all patients and their parents were briefed on the study’s risks and benefits, as well as the possibility of data use in future research projects.

The clinical assessment began with a thorough medical history (anamnesis) to identify any conditions relevant to the inclusion and exclusion criteria. Eligibility was further confirmed through clinical examination and radiographic evaluation. An extraoral physical examination was then performed to assess mandibular asymmetry, followed by a clinical evaluation for temporomandibular disorders (TMDs) based on the Diagnostic Criteria for Temporomandibular Disorders (DC/TMDs) [16,38,39]. GJH was assessed using the Beighton Score [40]. The radiological assessment included a panoramic radiograph to evaluate overall dental health and screen for exclusion criteria, and a posteroanterior cephalogram to assess MA. A single evaluator (L.M.V.) conducted each evaluation.

### 2.2. Mandibular Asymmetry by Postero-Anterior Cephalogram

Posteroanterior (PA) cephalograms were manually traced on acetate sheets, and anatomical landmarks were identified to construct an x–y coordinate system. The horizontal reference plane (*x*-axis) was defined by a line connecting the most medial point of the zygomaticofrontal suture and the lateral orbital rim bilaterally (ZL/ZR). The vertical reference plane (*y*-axis), referred to as the midfacial line, was established as a perpendicular line passing through the crista galli (Cg). MA was evaluated by quantifying menton deviation (Medev), defined as the absolute horizontal distance between the menton (Me) and the midfacial reference line [22]. Based on this measurement, patients were classified into two categories: symmetric (Medev < 4 mm) and asymmetric (Medev ≥ 4 mm).

### 2.3. GJH by Beighton Score (BS)

To evaluate BS of patients, five components were examined: (1) passive dorsiflexion and hyperextension of the fifth metacarpal joints beyond 90°, (2) passive apposition of the thumbs to the flexor aspects of the forearms, (3) passive hyperextension of the elbows beyond 10°, (4) passive hyperextension of the knees beyond 10°, and (5) active forward flexion of the trunk with fully extended knees, allowing the palms to rest flat on the floor. The elements 1 to 4 can score a maximum of 2 points each, as they are performed bilaterally (1 point for each hypermobile joint), while the last element has a maximum score of 1 point [40]. Therefore, the BS of participants ranges from 0 to 9 points. Patients were classified into the GJH-positive group if BS ≥ 4 and the GJH-negative group if BS < 4.

### 2.4. TMD by DC/TMD

All participants were clinically examined following the standardized procedures of Axis I of the DC/TMD, with diagnostic classification performed according to the DC/TMD decision tree [16,38,39]. Individuals presenting with any TMD diagnosis were categorized as TMD, while those without clinical signs or symptoms consistent with TMD were classified as No-TMD.

### 2.5. Statistical Analysis

The required sample size was calculated using G*Power software (version 3.1.9.7 for Windows, University of Düsseldorf, Düsseldorf, Germany) [41], based on an expected medium effect size (w = 0.30) [42] for the chi-square test, with an alpha level of 0.05 and a statistical power of 80%. The calculation indicated a minimum of 67 participants to detect significant associations among MA, GJH, and TMD. The final sample included 74 participants, thus exceeding the minimum requirement and ensuring adequate statistical power.

The collected data were analysed using standard descriptive statistics and presented as frequencies and percentages. The chi-square test was employed to assess the association between MA, GJH, and the presence of TMD. A *p*-value < 0.05 was considered statistically significant. For each significant chi-square result, Cramér’s V was calculated to determine the effect size and assess the strength of association, with interpretation as follows: small (0.10–0.30), moderate (0.30–0.50), and large (>0.50) [42]. Both *p*-values and effect sizes are reported to provide a more complete assessment of the findings. All statistical analyses were conducted using SPSS software (version 24.0; IBM Corp., Armonk, NY, USA).

## 3. Results

A total of 103 patients were evaluated. After applying inclusion and exclusion criteria, the final sample resulted in 74 patients, 19 males (25.68%), and 55 females (74.32%) (mean age 12.7 ± 2.16 years).

The GJH-positive group included 41 subjects, of whom 29 were females (70.7%) and 12 were males (29.3%). The GJH-negative group consisted of 33 subjects, comprising 26 females (78.8%), and 7 males (21.2%). No statistically significant association was found between the presence of GJH and sex (*p* > 0.05).

The analysis of TMD prevalence showed that 52 patients (70.3%) in the overall sample presented clinical signs and symptoms of TMD. Among them, 35 subjects (85.4%) in the GJH-positive group were diagnosed with TMD. A statistically significant association was identified between GJH and TMD, but this association strength is relatively weak (χ^2^ = 10.029; *p* = 0.002; Cramer’s V = 0.1). While evaluating the correlation between GJH and MA, 28 patients (68.3%) in the GJH-positive group were found to be asymmetric, compared to only 15 patients (45.5%) in the GJH-negative group. A statistically significant correlation was also observed between GJH and MA (χ^2^ = 3.988; *p* = 0.041; Cramer’s V = 0.1). (Table 1).

An analysis of the relationship between TMD and MA revealed that 71.2% of TMD patients had MA. The data showed a strong and statistically significant association between TMD and MA (χ^2^ = 12.229; *p* < 0.001; Cramer’s V = 0.3), which corresponds to a moderate effect size according to conventional thresholds. This indicates that the association, while not large, is of sufficient magnitude to be potentially meaningful in clinical contexts, particularly when considered alongside other risk factors (Table 2).

The relationship between MA, GJH, and TMD showed clear patterns in this sample. Among participants without GJH, MA was significantly more common in those diagnosed with TMD (χ^2^ = 5.241; *p* = 0.022; Cramer’s V = 0.1), indicating a small effect size.

A similar trend was observed in the GJH-positive group, although the association reached only marginal significance (χ^2^ = 3.967; *p* = 0.046; Cramer’s V = 0.1), which also represents a small effect size. This finding indicates that, in hypermobile individuals, the relationship between MA and TMD is weak (Table 3).

## 4. Discussion

This cross-sectional study was conducted to explore the relationship between MA, GJH, and TMD in pre-orthodontic growing individuals. The findings revealed a statistically significant relationship between MA and the presence of TMD, highlighting asymmetry as a potentially structural factor in the development of TMD. This association was further intensified in the presence of GJH, suggesting a risk factor when systemic and craniofacial vulnerabilities coexist.

The notably high prevalence of TMD within the study population, especially among individuals exhibiting GJH, suggests that joint hypermobility may play a significant role in compromising the stability of the TMJ. This instability likely increases the joint’s vulnerability to functional impairments and pathologies. The association between GJH and TMD has been extensively examined in the literature, with numerous studies highlighting a clear link between the two conditions [43,44,45,46]. Specifically, research indicates that GJH may serve as a risk factor or predisposition for TMD development, as individuals with generalized hypermobility tend to show a higher incidence of TMD compared to non-hypermobile control groups [46,47]. The underlying mechanisms proposed to explain this relationship center on the biomechanical and structural characteristics of hypermobile joints. In GJH, increased laxity of connective tissues and ligaments may reduce the mechanical support and resilience of the TMJ, making it more susceptible to abnormal loading, displacement, and eventual dysfunction. Consequently, hypermobile individuals may experience a greater frequency of disc displacements (DDs) and related symptoms, particularly during adolescence—a critical period marked by rapid growth and musculoskeletal development. Indeed, evidence suggests that patients with GJH often present with more severe forms of disc displacement, which may intensify and progress over time as the individual matures through adolescence [35,37].

Furthermore, the progression of TMD symptoms in hypermobile patients during adolescence underscores the potential cumulative impact of joint laxity combined with developmental factors. As the musculoskeletal system changes, the compromised stability may exacerbate functional disorders of the TMJ, leading to worsening clinical manifestations. This progression highlights the importance of early identification and monitoring of hypermobile individuals, particularly during growth periods when intervention may be most effective in preventing long-term dysfunction [35,37].

Clinically, understanding the interplay between GJH and TMD can inform both diagnostic and therapeutic approaches. Screening for joint hypermobility in patients presenting with TMD symptoms could improve risk stratification and personalized treatment planning. Therapeutic strategies might need to emphasize stabilization techniques, proprioceptive training, and cautious management of joint loading in hypermobile individuals to mitigate symptom progression and improve quality of life.

Overall, these findings support the hypothesis that GJH could be a factor in the etiology and severity of TMD, reinforcing the need for further longitudinal studies to elucidate the pathophysiological pathways and optimize clinical care for this at-risk population. Mandibular asymmetry alone was also significantly associated with TMD, regardless of GJH status, and the correlation between TMD and MA is a topic widely discussed in the literature [20,21,48,49]. This finding highlights the significant role of craniofacial morphology in TMJ health. Notably, even in the absence of GJH, alterations in mandibular alignment may lead to functional imbalances, muscular overload, or asymmetric joint loading—factors that can progressively contribute to the onset of pain, joint noises, or limitations in mandibular movement. Our data indicate that, although GJH may function as a risk modifier, skeletal asymmetry emerges as an associated factor in TMD development.

The function and morphology of the TMJ were associated with facial asymmetry, particularly in cases involving mandibular asymmetry [21,22]. Morphological differences between the right and left TMJs in patients with MA may reflect anatomical alterations that could be associated with TMD [22]. Such condylar changes, particularly a reduction in condylar height, can result in shortening on the side of displacement, thereby contributing to the development or accentuation of facial asymmetry [21]. Given this complex interplay, establishing a clear cause-and-effect relationship between FA and TMDs remains challenging.

It is important to acknowledge the limitations of this study. The sample size, although sufficient to detect significant associations, limits the generalizability of the results. Additionally, the cross-sectional design precludes any conclusions about causality. Future longitudinal studies are needed to determine whether facial asymmetry and GJH precede the development of TMD or emerge as consequences of ongoing dysfunction. It would also be beneficial to investigate how these relationships evolve with growth and whether early intervention can mitigate long-term risks.

The strength of the discovered correlations is another limitation. The effect size analysis revealed that while several relationships reached statistical significance, most associations were of small magnitude, with only a few reaching a moderate level. Despite being statistically significant, this suggests that some correlations may not be clinically significant [50]. Therefore, these findings should be interpreted with caution, and future research should attempt to replicate them in larger samples to investigate if stronger correlations may be identified in different demographics or longitudinal settings. Despite these limitations, this study contributes valuable insight into the interplay between structural and systemic factors in TMD pathogenesis. By recognizing facial asymmetry as a key variable, especially when accompanied by GJH, clinicians can better identify patients at risk and adopt a more personalized, preventive approach to care.

When considering the clinical implications, these findings support the importance of a comprehensive evaluation of both facial structure and GHJ in children and adolescents. Orthodontists and paediatric dentists are often the first to detect subtle asymmetries or signs of joint instability. While effect size analysis showed that most associations were small, the moderate association observed between TMD and MA suggests that identifying and monitoring mandibular asymmetry may be particularly relevant for risk assessment. Early recognition of such features, especially when combined with other risk factors, may allow for targeted monitoring or timely intervention, potentially preventing the progression of dysfunction into chronic or painful conditions.

Further research with larger samples and longitudinal designs is warranted to clarify causal relationships and to explore whether early identification of asymmetry could inform preventive or therapeutic strategies in at-risk populations.

## 5. Conclusions

Mandibular asymmetry emerged as a key factor associated with the presence of TMD, particularly when combined with GHJ. These findings indicate that mandibular asymmetric alone is significantly related to the occurrence of TMD, regardless of joint hypermobility status. However, the risk appears to be amplified in individuals who also present with GJH. Effect size analysis indicated that most associations were small, with only the one between TMD and MA reaching a moderate level. This highlights the importance of evaluating statistical significance in the context of effect size to better assess clinical relevance.

## Figures and Tables

**Table 1:** Relationship between GJH and TMD and between GJH and MA.

		TMD				Mandibular Asymmetry			
		No-TMD N (%)	TMD N (%)	Total N (%)	*p*-Value (Cramér’s V Values	Symmetric N (%)	Asymmetric N (%)	Total N (%)	*p*-Value (Cramér’s V Value)
GJH	GJH-negative	16 (48.5%)	17 (55.5%)	33 (100%)	0.002 * (0.1)	18 (54.5%)	15 (45.5%)	33 (100%)	0.041 * (0.1)
	GJH-positive	6 (14.6%)	35 (85.4%)	41 (100%)		13 (31.7%)	28 (68.3%)	41 (100%)	
Total		22 (29.7%)	52 (70.3%)	74 (100%)		31 (41.9%)	43 (58.1%)	74 (100%)	

N: count; * chi-square test: *p*-value < 0.05 indicates statistical significance.

**Table 2:** Relationship between TMD and MA.

		Mandibular Asymmetry		Total N (%)	*p*-Value (Cramér’s V Value)
		Symmetric N (%)	Asymmetric N (%)		
TMD	No-TMD	16 (72.7%)	6 (27.3%)	22 (100%)	0.001 * (0.3)
	TMD	15 (28.8%)	37 (71.2%)	52 (100%)	

N: count; * chi-square test: *p*-value < 0.05 indicates statistical significance.

**Table 3:** Relationship between GJH, TMD, and MA.

		Mandibular Asymmetry		Total N (%)	*p*-Value (Cramér’s V Values)
		Symmetric N (%)	Asymmetric N (%)		
GJH-negative	No-TMD	12 (75.0%)	4 (25.0%)	16 (100%)	0.022 * (0.1)
	TMD	6 (35.3%)	11 (64.7%)	17 (100%)	
GJH-positive	No-TMD	4 (66.7%)	2 (33.3%)	6 (100%)	0.046 * (0.1)
	TMD	9 (25.7%)	26 (74.3%)	35 (100%)	

N: count; * chi-square test: *p*-value < 0.05 indicates statistical significance.

## Data Availability

The data presented in this study is available upon request from the corresponding author. The data is not publicly available due to privacy and ethical restrictions.

## Abbreviations

The following abbreviations are used in this manuscript:

MA	Mandibular asymmetry
GHJ	Generalized Joint Hypermobility
TMD	Temporomandibular Disorders
TMJ	Temporomandibular Joint
BS	Beighton Score
